# Multimodality radiomics for tumor prognosis in nasopharyngeal carcinoma

**DOI:** 10.1371/journal.pone.0298111

**Published:** 2024-02-12

**Authors:** Sararas Khongwirotphan, Sornjarod Oonsiri, Sarin Kitpanit, Anussara Prayongrat, Danita Kannarunimit, Chakkapong Chakkabat, Chawalit Lertbutsayanukul, Sira Sriswasdi, Yothin Rakvongthai

**Affiliations:** 1 Department of Radiological Technology and Medical Physics, Faculty of Allied Health Sciences, Chulalongkorn University, Bangkok, Thailand; 2 Chulalongkorn University Biomedical Imaging Group, Department of Radiology, Faculty of Medicine, Chulalongkorn University, Bangkok, Thailand; 3 Division of Radiation Oncology, Department of Radiology, King Chulalongkorn Memorial Hospital, Bangkok, Thailand; 4 Division of Radiation Oncology, Department of Radiology, Faculty of Medicine, Chulalongkorn University, Bangkok, Thailand; 5 Center for Artificial Intelligence in Medicine, Research Affairs, Faculty of Medicine, Chulalongkorn University, Bangkok, Thailand; 6 Center of Excellence in Computational Molecular Biology, Chulalongkorn University, Bangkok, Thailand; 7 Division of Nuclear Medicine, Department of Radiology, Faculty of Medicine, Chulalongkorn University, Bangkok, Thailand; University of Pisa, ITALY

## Abstract

**Background:**

The prognosis of nasopharyngeal carcinoma (NPC) is challenging due to late-stage identification and frequently undetectable Epstein-Barr virus (EBV) DNA. Incorporating radiomic features, which quantify tumor characteristics from imaging, may enhance prognosis assessment.

**Purpose:**

To investigate the predictive power of radiomic features on overall survival (OS), progression-free survival (PFS), and distant metastasis-free survival (DMFS) in NPC.

**Materials and methods:**

A retrospective analysis of 183 NPC patients treated with chemoradiotherapy from 2010 to 2019 was conducted. All patients were followed for at least three years. The pretreatment CT images with contrast medium, MR images (T1W and T2W), as well as gross tumor volume (GTV) contours, were used to extract radiomic features using PyRadiomics v.2.0. Robust and efficient radiomic features were chosen using the intraclass correlation test and univariate Cox proportional hazard regression analysis. They were then combined with clinical data including age, gender, tumor stage, and EBV DNA level for prognostic evaluation using Cox proportional hazard regression models with recursive feature elimination (RFE) and were optimized using 20 repetitions of a five-fold cross-validation scheme.

**Results:**

Integrating radiomics with clinical data significantly enhanced the predictive power, yielding a C-index of 0.788 ± 0.066 to 0.848 ± 0.079 for the combined model versus 0.745 ± 0.082 to 0.766 ± 0.083 for clinical data alone (*p*<0.05). Multimodality radiomics combined with clinical data offered the highest performance. Despite the absence of EBV DNA, radiomics integration significantly improved survival predictions (C-index ranging from 0.770 ± 0.070 to 0.831 ± 0.083 in combined model versus 0.727 ± 0.084 to 0.734 ± 0.088 in clinical model, *p*<0.05).

**Conclusions:**

The combination of multimodality radiomic features from CT and MR images could offer superior predictive performance for OS, PFS, and DMFS compared to relying on conventional clinical data alone.

## Introduction

Nasopharyngeal carcinoma (NPC) is a type of malignancy that is more common in specific parts of the world, such as East Asia and Africa [1]. The identification of this disease can be challenging due to its vague symptoms, which may include a painless swelling or growth on either side of the neck’s rear, a result of lymph node enlargement from metastasis. The illness can cause a variety of symptoms, including sore throat, difficulty breathing, speaking, and hearing. Additional potential signs of NPC encompass facial discomfort or lack of sensation, vision impairment, and repeated ear infections [2, 3]. Reliable algorithms for prediction are essential for improving therapy strategies and patient prognosis due to the complexity of NPC. The primary treatment strategy is intensity-modulated radiation therapy (IMRT), which can be used with chemotherapy to reduce the size of the tumor and increase the effectiveness of radiation [4]. To ensure the implementation of the most effective treatment plan, it is crucial to employ precise prognostic instruments that can forecast the patient’s reaction to therapy, as well as the likelihood of relapse or disease advancement.

Plasma Epstein-Barr virus (EBV) DNA is a commonly used biomarker for early detection, prognosis, and monitoring of treatment outcomes in NPC [5–9], often used alongside the clinical staging protocol developed by the American Joint Committee on Cancer (AJCC) [7, 10–12]. However, it is worth noting that EBV DNA levels are not detectable in up to 40% of non-Chinese patients [13]. This highlights the need for more efficient biomarkers to improve the accuracy of prognostic assessments.

Radiomics is a rapidly-growing field that utilizes advanced mathematical methods to extract extensive quantitative data from medical images [14–19]. In the realm of head and neck oncology, its noninvasive applications are particularly promising for differentiating benign from malignant lesions [20]. A crucial task given the complex anatomy of the head and neck where traditional biopsy may be challenging. Radiomic signatures are not limited to identification; they have potential in correlating with treatment responses [21, 22], prognostic outcomes [23, 24], and even genetic expressions [25]. Such insights are invaluable in customizing treatment for head and neck cancer patients. A variety of imaging modalities, including [26], MRI [27], PET/CT [28], and ultrasound [29], are instrumental in radiomics research. They provide a foundation for extracting radiomic features, which are essential in the ongoing research to develop noninvasive biomarkers for these cancers.

For NPC patients, it is a standard practice to perform computed tomography (CT) and magnetic resonance (MR) simulations prior to radiotherapy to collect data for treatment planning [30, 31]. Building upon our previous research, which established a prognostic model using CT images alone [32], this study attempted to enhance model performance by integrating MRI data. We hypothesized that MRI can reveal additional tumor characteristics, particularly soft tissue anomalies, which are not as discernible on CT. By enriching our model with these multidimensional radiomic features, we anticipated a marked improvement in its prognostic power. Our goal was to construct a more comprehensive prognostic model by combining clinical factors with a multimodal radiomic approach, incorporating both CT and MRI data, to predict key survival outcomes for NPC patients, including overall survival (OS), progression-free survival (PFS), and distant metastasis-free survival (DMFS). By doing so, we expected to provide an improved tumor prognostic tool, which could lead to more personalized and effective treatment strategies, ultimately advancing NPC patient management.

## Materials and methods

### Data collection

Medical data of patients with NPC, between October 2010 and January 2019, at King Chulalongkorn Memorial Hospital, Bangkok, Thailand, were retrospectively collected (accessed on September 30, 2021). The patients were partially involved in a previously published randomized study comparing the intensity-modulated radiation treatment (IMRT) technique between sequential boost and simultaneous integrated boost technique [33], as well as in a radiomics study for NPC tumor prognosis using only CT images [32]. The following eligibility requirements had to be met for patients with newly diagnosed NPC to participate in the study: (a) absence of distant metastasis; (b) at least three years of follow-up; (c) encountering CT with contrast medium and T1W- and T2W-MRI simulation; (d) receiving IMRT with chemotherapy; and (e) having pre-treatment plasma Epstein-Barr virus (EBV) DNA level.

Demographic and clinical information were collected, including age, sex, and tumor stage, and the patients were then restaged based on the 8th edition cancer staging of AJCC. All patients were treated based on the severity of their disease. In detail, those with T1 or less and negative nodal disease were treated with IMRT alone while patients with ≥ T2 and/or positive nodal disease received IMRT with concurrent weekly 40 mg/m^2^ cisplatin up to seven cycles, followed by adjuvant chemotherapy comprised of 80 mg/m^2^ cisplatin and 1000 mg/m^2^/24 hr of 5-fluorouracil (5-FU) administered continuously for 96 hours at 4-week intervals for three cycles. The institutional review board of the Faculty of Medicine, Chulalongkorn University approved this retrospective study (IRB no. 672/64). The need for written informed consent was waived by the ethics committee. All the data were anonymized before analysis, and all the methods were performed in accordance with the relevant guidelines and regulations.

### Imaging protocols of CT and MRI

CT images acquired from the CT scanners (Somatom Definition AS, Siemens Medical Solutions, Erlangen, Germany and Brilliance Big Bore, Philips Healthcare, The Netherlands) used for radiotherapy treatment planning simulations were collected. The scanning parameters were as follows: 120 kVp, 325 mAs, FOV 600x600 mm, and slice thickness of 2 and 3 mm. Image acquisition was performed 59 seconds after injecting 75 ml of nonionic iodinated contrast medium (iohexol 300mgI/mL; Omnipaque 300, GE Healthcare). Magnetic resonance (MR) images were acquired on a 1.5 T MRI simulator (Signa HDxt, GE Medical systems, Chicago, United states) at Division of Radiation Oncology. The standard protocol for the nasopharyngeal study was used, including axial T1-weighted images (repetition time (TR): 740 ms; echo time (TE): 8.3ms) and axial T2-weighted images (repetition time (TR): 5395 ms; echo time (TE): 68.6 ms), with FOV 24 cm and slice thickness of 4 mm.

### Tumor segmentation

Gross tumor volume (GTV) was contoured on CT images using Eclipse software (Varian Medical Systems, Palo Alto, CA, USA) by one of three board-certified radiation oncologists, specializing in head-and-neck cancer, with varying levels of experience (19, 8, and 4 years post board certification). To facilitate the CT-MR image alignment, the region of interest (ROI) encompassing the GTV on CT was mapped to the corresponding MR images using 3D-slicer software version 4.11. The mapping process involved identifying anatomical landmarks on both the CT and MR images, as well as utilizing the registration location produced during the radiation oncologist’s treatment planning process to ensure accurate registration. The resulting MR images with the mapped ROI were then used for further analysis.

To assess for interobserver variability, we randomly sampled 22 patients out of the total sample size. For each of these 22 patients, the GTV was independently contoured by the other two board-certified radiation oncologists who were blinded to each other and the original GTV contours. These additional radiation oncologists utilized the same imaging software and datasets as the primary radiation oncologist who initially contoured the GTV.

### Model development and assessment

#### (A) Construction of the radiomic model

The features were extracted via PyRadiomics version 2.0.0 [34] using the data from CT and MR images along with their corresponding ROIs. Prior to radiomic feature extraction, the original CT and MR images were resampled to a voxel size of 0.5 cm^3^ to ensure spatial consistency. A total of 842 radiomic features were obtained from the GTV contour of each imaging modality, including first-order features (18), shape features (14), texture features (74), and wavelet features (736). This resulted in 2526 radiomic features from three images (contrast-enhanced CT, T1W-MRI, and T2W-MRI) being extracted per patient. The process of extracting radiomic features was carried out using the standard configurations in the software package.

To identify the most reliable and informative radiomic features for prediction, we implemented a dual-step strategy; an interobserver variability test and a univariate analysis. Initially, to assess the stability of the radiomic features, we carried out an interobserver variability examination on a subset of the dataset. To do this, we randomly selected 22 patients and tasked three distinct radiation oncologists with the manual delineation of the ROI. Following this, we extracted radiomic features from the three ROI sets (contrast-enhanced CT, T1W-MRI, T2W-MRI) and computed the intraclass correlation coefficient (ICC) for each feature to measure the level of agreement among observers. Features with poor interobserver agreement (ICC < 0.50) were excluded from further analysis [35].

Subsequently, we performed a univariate Cox proportional hazards regression analysis on the remaining radiomic features to evaluate their association with the clinical outcomes, which was OS, PFS, and DMFS. For this analysis, the CoxPHSurvivalAnalysis function from the scikit-survival library in Python was utilized. Features that ranked in the top 20% in terms of the C-index were retained for additional analysis.

To construct our radiomic model, we first applied recursive feature elimination with cross-validation (RFECV), utilizing a single iteration of five-fold cross-validation to discern the most significant features from the univariate analysis. This process aimed to identify a feature set that maximized the Concordance Index (C-index), selecting the minimal number of features necessary for the highest prognostic accuracy. After RFECV, L2 regularization (ridge regression) with a regularization strength of C = 1 was employed to fine-tune the feature coefficients and prevent overfitting. To assure the reproducibility and stability of our model, we further subjected it to a rigorous validation using a five-fold cross-validation procedure, which was repeated 20 times with a variety of random seeds. The predictive performance of the model was consistently evaluated using the C-index. The models were independently constructed, resulting in CT-based radiomic, MRI-based radiomic, and CT+MRI-based radiomic models.

#### (B) Construction of the clinical model

We also created a clinical model, which was a based-line model, for predicting survival outcomes to compare with the radiomic model. Clinical variables including age, sex, T stage, N stage, overall stage, and Epstein-Barr virus (EBV) value were included. A binary sequence encoding was adopted for the T stage, N stage, and overall stage to effectively capture the ordinal nature of disease progression. Each stage was represented by a binary sequence reflecting its hierarchical position (e.g., T-stage 1 as 0 0 0, and T-stage 4 as 1 1 1). The EBV value was transformed into a binary feature using a threshold value of 2300 copies/ml [13]. The similar strategy to the radiomic model to identify the most relevant clinical features was used. Specifically, ridge (L2) regularization with five-fold cross validation strategy with 20 iterations was used to evaluate the performance and generalizability. The model that initially excluded EBV value was also constructed.

#### (C) Construction of combined model

Three more comprehensive models—clinical+CT-based radiomic, clinical+MRI-based radiomic, and clinical+CT+MRI-based radiomic—were created by combining the chosen clinical and radiomic features. The similar strategy, which was L2 Cox proportional hazards regression with 20 iterations of five-fold cross-validation procedure, was used.

### Performance assessment of constructed models

To evaluate the models’ performance, we used Harrell’s C-index, a commonly used statistical measure for assessing the discriminative power of survival models. The C-index was calculated for each model on the training and test set of each fold throughout the cross-validation process. A sign test, which assesses the statistical significance of the difference in C-index values between two models, was also used to assess the performance of the radiomic, clinical, and combination models. Multiple comparison correction was carried out using the Benjamini-Hochberg method. All statistical calculations were performed in Python, and a p-value of 0.05 was considered statistically significant.

## Results

### Patient demographics

The study cohort was composed of 183 patients diagnosed with NPC. The majority of the participants were male (78.14%). The median age was 50 years with an interquartile range (IQR) of 42.5 to 57.5 years **(Table 1)**. A significant portion of the patients were in stage III (50.82%) or IV (33.88%) of the disease. Each participant received definitive radiation therapy in combination with chemotherapy. The median follow-up duration was 54.5 months with an IQR of 41.9 to 65.3 months. At 3 years after starting treatment, 29 patients had died (from any cause), 45 patients had experienced disease progression or recurrence, and 42 patients had developed distant metastases.

**Table 1:** Patient demographics.

Characteristics	n = 183 (%)	p-value
Median age (years) (IQR)	50 (42.5 to 57.5)	
Sex		<0.001
Male	143 (78.14)	
Female	40 (21.86)	
T classification		0.01
T1	51 (27.87)	
T2	59 (32.24)	
T3	45 (24.59)	
T4	28 (15.30)	
N classification		<0.001
N0	10 (5.46)	
N1	42 (22.95)	
N2	90 (49.18)	
N3	41 (22.40)	
Stage group		<0.001
I	3 (1.64)	
II	25 (13.66)	
III	93 (50.82)	
IVA	62 (33.88)	
Pretreatment plasma EBV DNA level		0.71
undetectable or ≤ 2300 copies/ml	94 (51.37)	
> 2300 copies/ml	89 (48.63)	
median value (copies/ml) (IQR)	9720 (5160 to 23400)	
Pathological classification		<0.001
undifferentiated non keratinizing carcinoma	148 (80.87)	
differentiated non keratinizing carcinoma	28 (15.30)	
keratinizing squamous cell carcinoma	3 (1.64)	
undifferentiated carcinoma and other	4 (2.19)	

### Inter-observer variability effect over radiomics

The accuracy and reliability of radiomics features depend on the quality and consistency of the tumor delineation process. Therefore, we examined the influence of interobserver variability on the radiomic features by measuring their intraclass correlation coefficient (ICC) calculated from the GTV contours drawn by three independent radiation oncologists. We varied the ICC cutoff threshold to assess the impact of different levels of interobserver variability on the number of radiomic features that passed the criterion and the final prognosis prediction performance. Specifically, we used cutoff values of 0.9, 0.75, and 0.5, which correspond to high, moderate and low levels of agreement [35], respectively. At these cutoffs, there were 235, 516, and 739 radiomic features that passed the thresholds. The steep drop in the number of features highlights the importance of careful tumor delineation and the need to consider robustness metrics such as interobserver variability when selecting radiomic features for use in prognostic models.

### Performance of constructed models

**(A) Radiomic model.** For OS, the combined model incorporating both CT and MRI-based radiomics achieved a higher C-index in the test set compared to models using radiomic features from just one data type. In the case of PFS, adding MRI features to the CT-based radiomics did not alter the performance; however, there was a statistically significant difference when compared to the MRI-only radiomic model (p<0.05). For DMFS, the combined model presented the highest predictive value, showing a statistically significant improvement (p<0.05) (**Table 2**).

**Table 2:** C-indices of clinical, radiomic, and combined models with Epstein-Barr Virus (EBV) value in overall survival (OS), progression-free survival (PFS), and distant metastasis-free survival (DMFS).

Outcome	Model name	Training set	Test set	Number of features
OS	Clinical	0.808 (±0.023)	0.750 (±0.110)	8
	CT	0.849 (±0.016)	0.822 (±0.070)	9
	MRI	0.825 (±0.017)	0.796 (±0.080)	9
	CT+MRI	0.879 (±0.014)	0.827 (±0.067)	18
	Clinical +CT	0.893 (±0.016)	0.827 (±0.084)	17
	Clinical +MRI	0.894 (±0.016)	0.828 (±0.085)	17
	Clinical +CT+MRI	0.919 (±0.014)	0.848 (±0.079)	26
PFS	Clinical	0.788 (±0.020)	0.766 (±0.083)	6
	CT	0.721 (±0.016)	0.722 (±0.072)	2
	MRI	0.704 (±0.017)	0.708 (±0.075)	1
	CT+MRI	0.721 (±0.016)	0.722 (±0.071)	3
	Clinical +CT	0.825 (±0.016)	0.801 (±0.068)	8
	Clinical +MRI	0.819 (±0.017)	0.796 (±0.069)	7
	Clinical +CT+MRI	0.826 (±0.016)	0.802 (±0.066)	9
DMFS	Clinical	0.765 (±0.019)	0.745 (±0.082)	4
	CT	0.710 (±0.017)	0.711 (±0.076)	2
	MRI	0.784 (±0.018)	0.766 (±0.067)	10
	CT+MRI	0.802 (±0.016)	0.779 (±0.064)	12
	Clinical +CT	0.805 (±0.016)	0.788 (±0.066)	6
	Clinical +MRI	0.844 (±0.016)	0.802 (±0.062)	14
	Clinical +CT+MRI	0.855 (±0.015)	0.811 (±0.062)	16

#### (B) Clinical model

The clinical model was formulated utilizing 15 input features, with its optimization contingent upon the selection of the optimal feature set. For OS prediction, a model derived from 8 clinical features was established. Likewise, PFS was modeled using 6 clinical features, while DMFS was based on four clinical features. Notably, recurring predictors across all three models included sex, T-stage 2 and above, N-stage 3, and EBV status, underscoring their potential prognostic significance for NPC (**S1 Fig**).

When the EBV value was initially excluded, there was a statistically significant decrease in the performance of the models for OS and PFS compared to the model with EBV value (*p* < 0.05) (**Tables 2 and 3**). Traditional clinical features such as sex, T-stage 2 and above were common predictors for NPC patients with undetectable EBV values. **(S4 Fig)**.

**Table 3:** C-indices of clinical, radiomic, and combined models without Epstein-Barr Virus (EBV) value in overall survival (OS), progression-free survival (PFS), and distant metastasis-free survival (DMFS).

Outcome	Model name	Training set	Test set	Number of features
OS	Clinical	0.779 (±0.026)	0.729 (±0.125)	7
	Clinical +CT	0.878 (±0.018)	0.820 (±0.080)	16
	Clinical +MRI	0.875 (±0.017)	0.808 (±0.084)	16
	Clinical +CT+MRI	0.906 (±0.015)	0.831 (±0.083)	25
PFS	Clinical	0.762 (±0.019)	0.727 (±0.084)	8
	Clinical +CT	0.807 (±0.015)	0.775 (±0.069)	10
	Clinical +MRI	0.801 (±0.016)	0.770 (±0.070)	9
	Clinical +CT+MRI	0.808 (±0.015)	0.775 (±0.068)	11
DMFS	Clinical	0.769 (±0.019)	0.734 (±0.088)	8
	Clinical +CT	0.811 (±0.016)	0.777 (±0.072)	10
	Clinical +MRI	0.844 (±0.014)	0.796 (±0.063)	18
	Clinical +CT+MRI	0.855 (±0.013)	0.801 (±0.061)	20

#### (C) Combined model

Integrating selected radiomic features from both CT and MRI with clinical features showed enhanced predictive performance. For OS prediction, the combined CT and MRI model with clinical features significantly outperformed the models using only CT or MRI radiomics with clinical variables (p < 0.05). Similarly, for DMFS, the integrated radiomics model displayed significant superiority over single-modality models (p < 0.05). For PFS, however, adding just one MRI-based radiomic feature didn’t result in a significant performance change. Further details, including the number of features used, was presented in **Table 2**.

When excluding the EBV value, combining both CT and MRI radiomics with clinical features still showed statistically improved predictive performance for OS and DMFS (p < 0.05) compared to single-modality models. All specifics can be found in **Table 3**.

### Model comparison

The comparison of the three model types—Radiomic, Clinical, and Combined models—shows that the performances of the constructed models vary considerably across different prediction tasks. For instance, in OS prediction, the radiomic model demonstrated superior predictive power compared to using traditional clinical features, with significantly higher C-index values ranging from 0.827 (±0.084) to 0.848 (±0.079) in the test set. In contrast, the clinical model achieved a C-index of only 0.750 (±0.110) in the test set.

**Fig 1:**
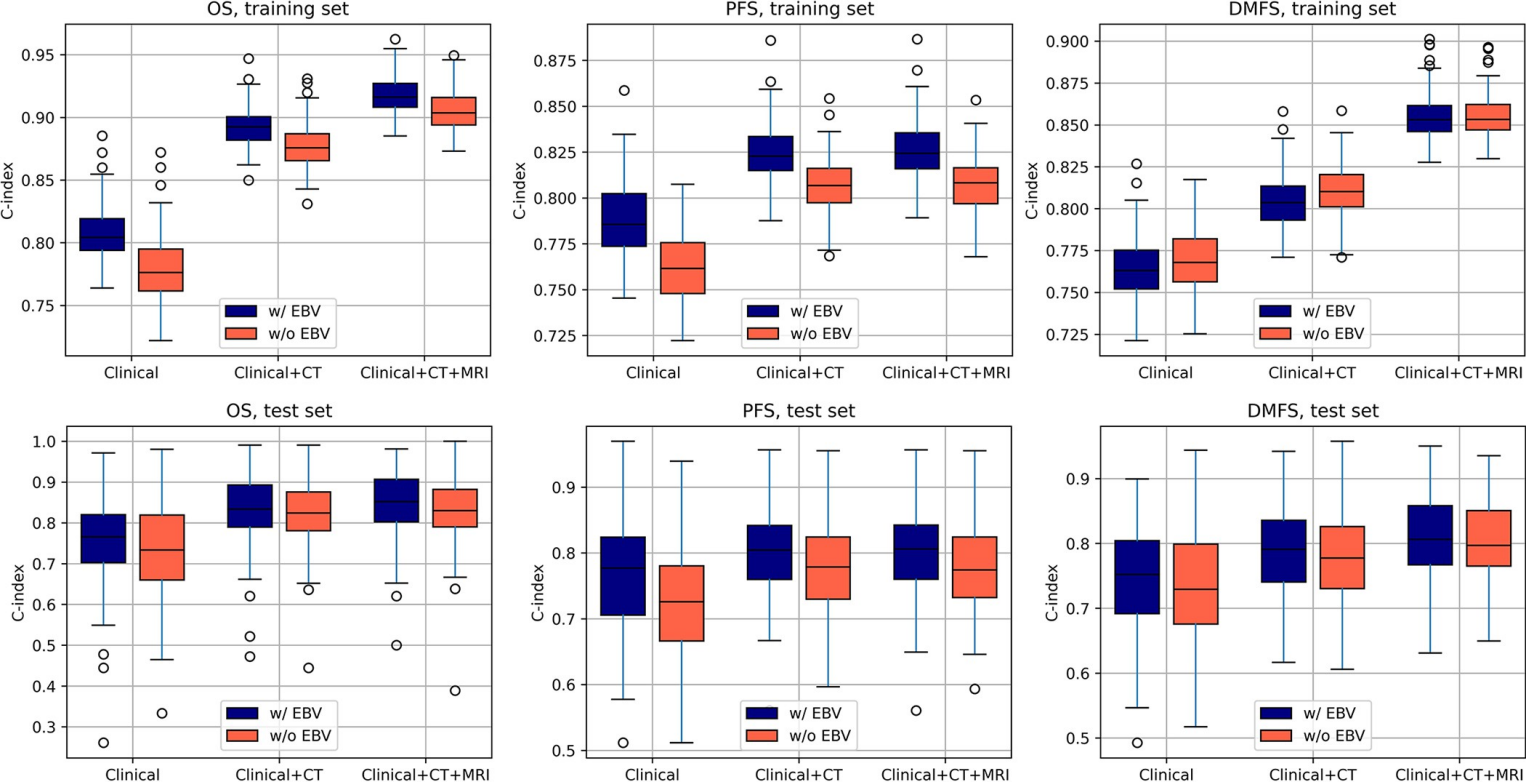
Boxplots of C-indices. (A) overall survival (OS), (B) progression-free survival (PFS), (C) distant metastasis-free survival (DMFS) in training set and (D) OS, (E) PFS, (F) DMFS in test set of the clinical, combination of clinical and CT-based radiomic, and combination of clinical and CT- and MRI-based radiomics models.

The predictive power was further pronounced when clinical features were combined with CT and MRI radiomic features in the combined model, which yielded a C-index of 0.848 (±0.079) in the test set when. Similarly, in DMFS prediction, the combination of CT and MRI radiomic features in the test set achieved a significantly higher C-index of 0.779 (±0.064) than the clinical model, which yielded a C-index of 0.745 (±0.082) (*p* < 0.05). Moreover, the performance improved even further when radiomics was combined with clinical features. The performance of the combined model ranged from 0.805 (±0.016) to 0.855 (±0.015) in the training set and 0.788 (±0.066) to 0.811 (±0.062) in the test set. In PFS, the radiomic models did not show superior performance compared to using only clinical features. However, the combination of radiomics with clinical features achieved the highest C-index, up to 0.826 (±0.016) in the training set and 0.802 (±0.066) in the test set, compared to the clinical model, which achieved a C-index of only 0.788 (±0.020) and 0.766 (±0.083) in the training and test sets, respectively. Overall, the integration of multimodal radiomic information improved model performance. As shown in **Fig 1**, the incorporation of radiomics with conventional clinical features provided more predictive information. In both the training and test sets, all models showed statistically significant differences (*p* < 0.05), except for the C-indices of PFS as compared to clinical+CT and clinical+CT+MRI in the test set.

In the model that did not include the Epstein-Barr virus (EBV) value, the integration of radiomic features from multimodality imaging with traditional clinical features also showed consistency with the model that included EBV. The combined model’s performance achieved higher C-indices in the test set, which was statistically higher than the clinical model (0.831 (±0.083) to 0.770 (±0.070) vs 0.734 (±0.088) to 0.727 (±0.084); p < 0.05, respectively).

**Fig 2:**
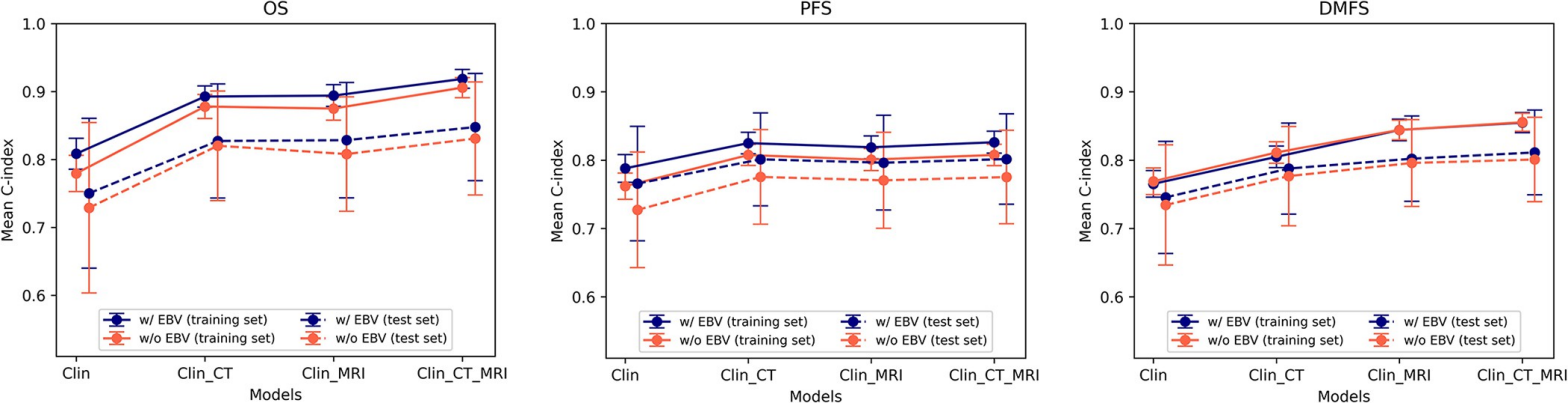
Line plots of C-indices of models with and without Epstein-Barr Virus (EBV) value. (A) overall survival (OS), (B) progression-free survival (PFS), and (C) distant metastasis-free survival (DMFS).

**Fig 3:**
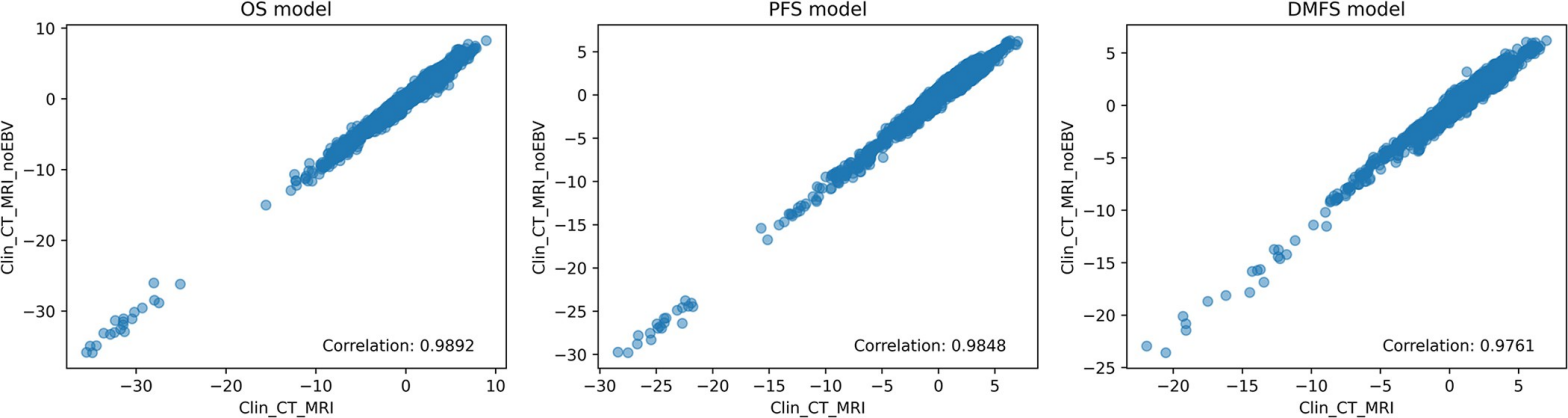
Scatter plots representing the correlation between the risk scores of the combined model. (A) Overall Survival (OS), (B) Progression-Free Survival (PFS), and (C) Distant Metastasis-Free Survival (DMFS) either with or without Epstein Barr Virus (EBV). The strong correlation in each plot underscores the similar predictive performance of the two models across all three survival outcomes.

When comparing models with and without the EBV value, the model that included EBV showed a higher predictive value than the model without EBV, with a statistically significant difference in OS and PFS prediction. Interestingly, for DMFS prediction, the clinical model and combined models newly constructed from features excluding the EBV value did not show a statistically significant difference when compared to the model constructed with the EBV value (*p* < 0.05) (**Fig 2**). In further analysis, we computed the Pearson correlation of predicted risk score between the combined models, incorporating clinical, CT, and MRI data, both with and without the inclusion of EBV. These models, noted for their superior prognostic performance, exhibited a very strong positive linear relationship, signified by a correlation coefficient exceeding 0.97 for all the outcomes (**Fig 3**), including OS, PFS, and DMFS. The robust correlation suggests that the model excluding the EBV value aligns closely with the model that includes EBV, indicating a comparable level of prediction between the two. The list of the selected features and their coefficient is shown in **S1–S6 Figs**.

## Discussion

Radiomics is an emerging field that has gained popularity due to its potential in various applications, such as diagnosis, prediction of treatment response, and prognosis. Although several studies have demonstrated the effectiveness of radiomics in predicting outcomes using a single modality, limited research has been conducted to investigate the predictive value of radiomics in multimodality, such as using PET/CT, PET/MRI, CT and MRI [36–38]. Specifically, to our knowledge, there was no study exploring the use of both CT and MR images for nasopharyngeal carcinoma (NPC). In this study, we reported the first and largest dataset used to build a predictive model for NPC using radiomics based on both pretreatment CT and MRI simulation. Our study aimed to investigate the potential of radiomic features extracted from CT and MRI images, in combination with traditional clinical features, to predict overall survival (OS), progression-free survival (PFS), and distant metastasis-free survival (DMFS) in patients diagnosed with NPC.

Our findings demonstrated that integrating both radiomic and clinical data together considerably increased the predictability when compared to using only radiomic or clinical features alone. In particular, the model’s performance increased when clinical variables and multimodal radiomics (CT and MRI) were included. This could be explained by the ability of radiomic features from CT and MRI, which provide details on structural and soft tissue characteristics, respectively, to capture hidden phenotypic information that might be neglected by the naked eyes alone. As a result, the predictive performance was improved as compared to using only conventional clinical features. Despite differences in the technique of model construction and the chosen radiomic features, our previous study, which concentrated solely at CT-based radiomics, showed that a radiomic signature had additional predictive gain when combined with conventional clinical features [32].

In the present study, radiomic features that were derived from MR images were added to the model to increase its performance. Our findings are consistent with a study by Li et al. [36], which showed superior performance when CT and MRI radiomic features were combined in determining therapy response in rectal cancer. The combination of both modalities showed better performance than using either modality alone. Additionally, He et al. [38] showed the potential of a multimodal (MRI/CT) radiomic model for prognostic prediction in resected hepatocellular carcinoma patients, with an improvement in prediction compared to using a single modality alone. Moreover, they investigated the potential of combining radiomic signature with clinical data, resulting in a C-index of 0.738 (0.575–0.901) for OS prediction and 0.704 (0.563–0.845) for disease-free survival (DFS) prediction in the validation cohort, which was higher than using models from either radiomic or clinical features alone.

The exclusion of the Epstein-Barr virus (EBV) value from the models presented an interesting scenario for discussion. The EBV value is a well-known biomarker for NPC, and its absence from the models may have potentially impacted their predictive performance. However, the results indicate that even without the EBV value, the models still maintained a significant level of predictive accuracy, albeit with a slight decrease. The combination of clinical and multimodality radiomics features continued to exhibit the strongest predictive performance across all outcomes (OS, PFS, DMFS) in both training and test set in the absence of EBV value. The Pearson correlation test of their predicted risk score was also carried out. This approach allowed us to quantitatively measure the degree of linear association between the two models, providing us a clear understanding of how closely the models align when predicting survival outcomes. The substantial correlation coefficient, exceeding 0.97, suggests a strong agreement between the models’ predictions, thereby reinforcing our finding that the absence of EBV information as well as patients with undetectable EBV value can be compensated by integrating clinical features with radiomics features derived from CT and MRI. The number of features required to construct the predictive models for PFS and DMFS increased when the EBV value was excluded. This could imply that the models needed to leverage more information to maintain their predictive power in the absence of the EBV value. Interestingly, for DMFS prediction, the combined model demonstrated no statistically significant difference between the models with and without the EBV value. This suggests that the model without EBV could potentially be used as a substitute for patients with undetectable EBV or for patients in centers that EBV results are not available, maintaining a similar level of predictive accuracy. This finding underscores the potential of radiomics in enhancing the precision of prognostic evaluations, even in the absence of certain clinical biomarkers. Nevertheless, further research is needed to validate these findings and to explore the potential of radiomics in improving the management of NPC patients.

One of the findings of this study revealed how interobserver variability impacts radiomic features, highlighting the importance of careful tumor delineation and the need to take this into account when selecting radiomic features to be included in prognostic models. The study showed that the accuracy and reliability of radiomic features depend on the quality and consistency of the tumor delineation. Therefore, it is crucial to reduce interobserver variability by training radiologists and using quality control procedures. The limitation of this study is that it is a retrospective study design and use of a dataset from a single institution. Future studies should explore the generalizability of these findings using external datasets.

## Conclusions

Our study demonstrates the possibility of enhancing prognostic prediction for NPC patients by combining radiomic features from both CT and MRI modalities with conventional clinical variables. This approach could have important clinical implications for decision-making in the management of nasopharyngeal cancer patients, particularly in the selection of appropriate treatment strategies and the identification of high-risk patients who may require closer monitoring.

## Supporting information

S1 Fig Clinical features and their coefficients in the clinical model for prediction. (A) overall survival (OS), (B) progression-free survival (PFS), and (C) distant metastasis-free survival (DMFS) in patients with nasopharyngeal carcinoma.
(PDF)

S2 Fig Clinical and CT-based radiomic features and their coefficients in the combined model for prediction. (A) overall survival (OS), (B) progression-free survival (PFS), and (C) distant metastasis-free survival (DMFS) in patients with nasopharyngeal carcinoma.
(PDF)

S3 Fig Clinical and CT- and MRI-based radiomic features and their coefficients in the combined model for prediction. (A) overall survival (OS), (B) progression-free survival (PFS), and (C) distant metastasis-free survival (DMFS) in patients with nasopharyngeal carcinoma.
(PDF)

S4 Fig Clinical features and their coefficients in the clinical model without Epstein-Barr Virus (EBV) value for prediction. (A) overall survival (OS), (B) progression-free survival (PFS), and (C) distant metastasis-free survival (DMFS) in patients with nasopharyngeal carcinoma.
(PDF)

S5 Fig Clinical and CT-based radiomic features and their coefficients in the combined model without Epstein-Barr Virus (EBV) value for prediction. (A) overall survival (OS), (B) progression-free survival (PFS), and (C) distant metastasis-free survival (DMFS) in patients with nasopharyngeal carcinoma.
(PDF)

S6 Fig Clinical and CT- and MRI-based radiomic features and their coefficients in the combined model without Epstein-Barr Virus (EBV) value for prediction. (A) overall survival (OS), (B) progression-free survival (PFS), and (C) distant metastasis-free survival (DMFS) in patients with nasopharyngeal carcinoma.
(PDF)
