# Supplementary figures and images for: Multimodality radiomics for tumor prognosis in nasopharyngeal carcinoma
Source: PLoS One. 2024 Feb 12;19(2):e0298111. doi: 10.1371/journal.pone.0298111 (PMC10861073; doi:10.1371/journal.pone.0298111)

S1 Fig

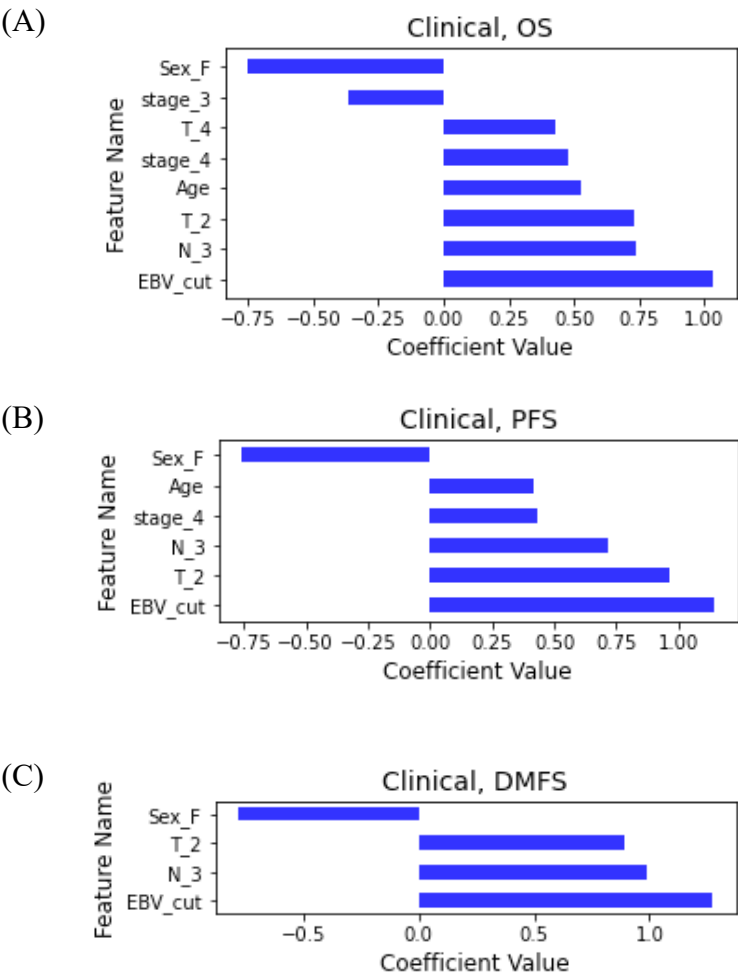

Supplement: S1 Fig — (A) overall survival (OS), (B) progression-free survival (PFS), and (C) distant metastasis-free survival (DMFS) in patients with nasopharyngeal carcinoma. (PDF) [file pone.0298111.s001.pdf]

S2 Fig

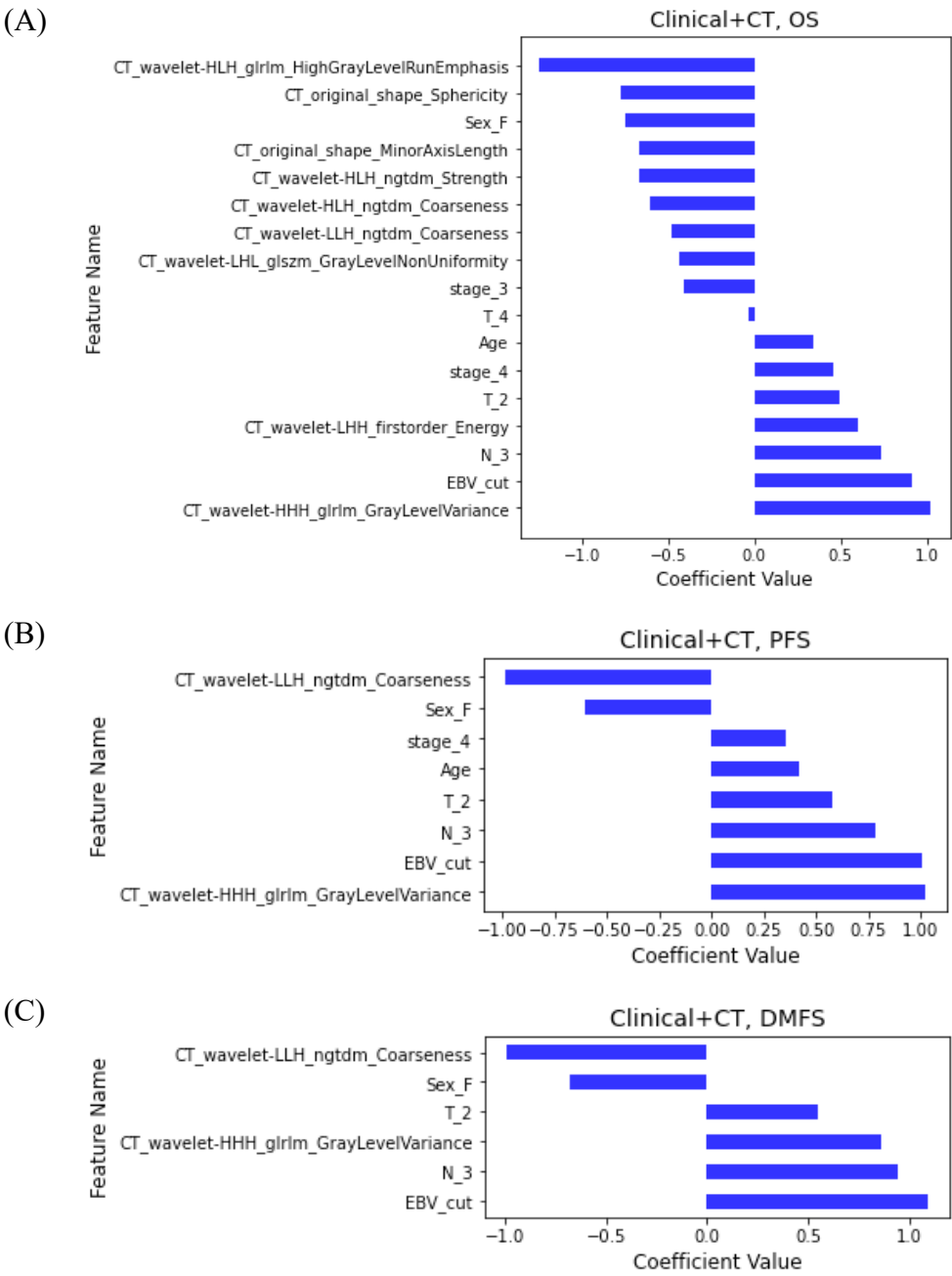

Supplement: S2 Fig — (A) overall survival (OS), (B) progression-free survival (PFS), and (C) distant metastasis-free survival (DMFS) in patients with nasopharyngeal carcinoma. (PDF) [file pone.0298111.s002.pdf]

S3 Fig

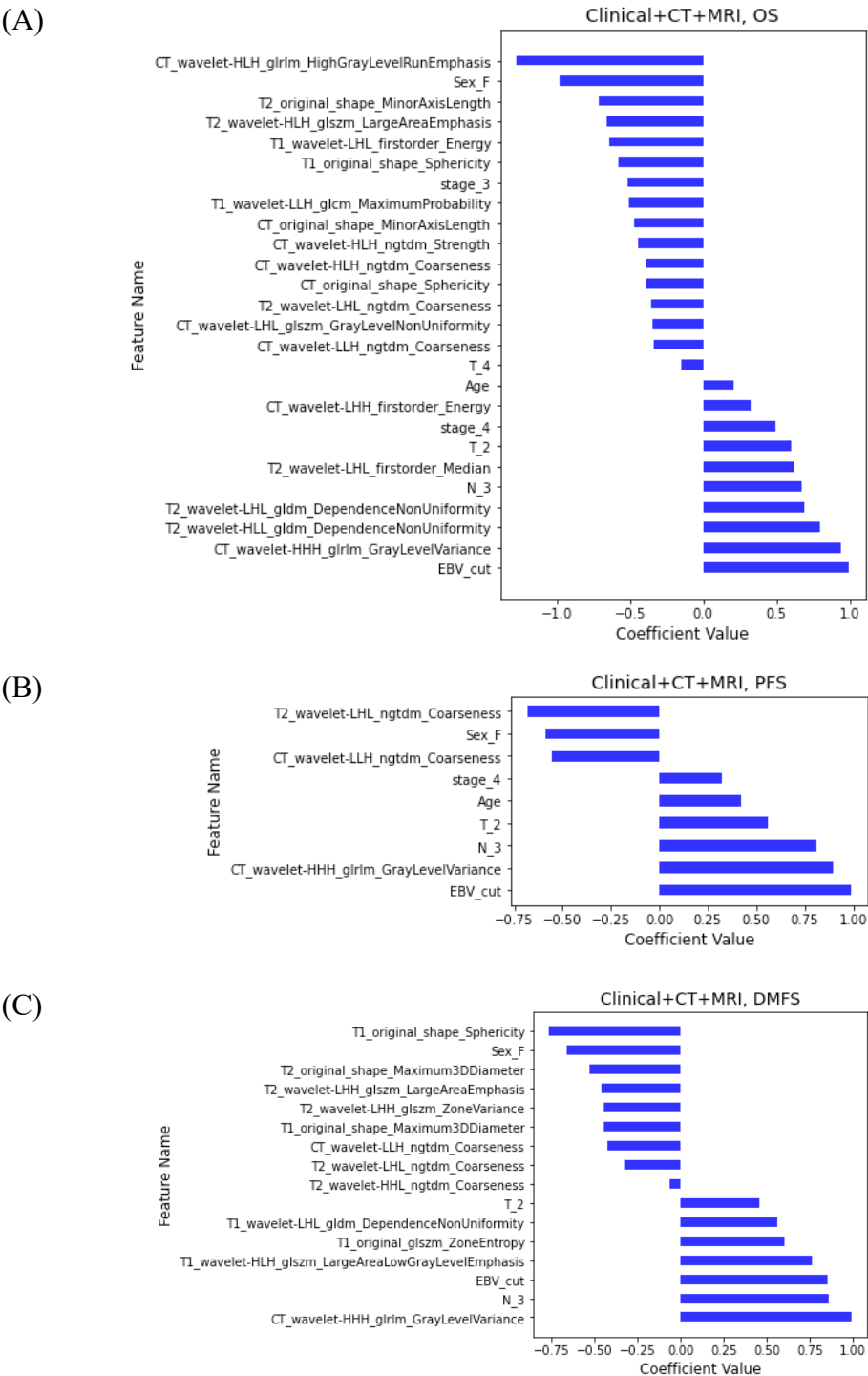

Supplement: S3 Fig — (A) overall survival (OS), (B) progression-free survival (PFS), and (C) distant metastasis-free survival (DMFS) in patients with nasopharyngeal carcinoma. (PDF) [file pone.0298111.s003.pdf]

S4 Fig

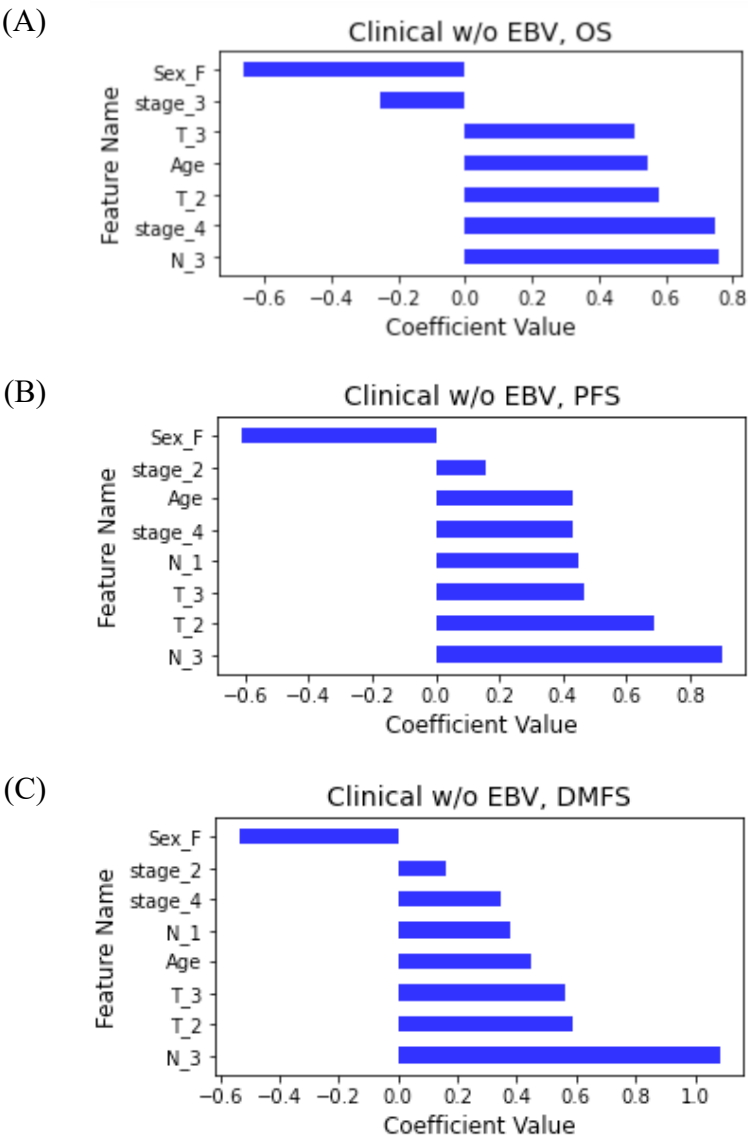

Supplement: S4 Fig — (A) overall survival (OS), (B) progression-free survival (PFS), and (C) distant metastasis-free survival (DMFS) in patients with nasopharyngeal carcinoma. (PDF) [file pone.0298111.s004.pdf]

S5 Fig

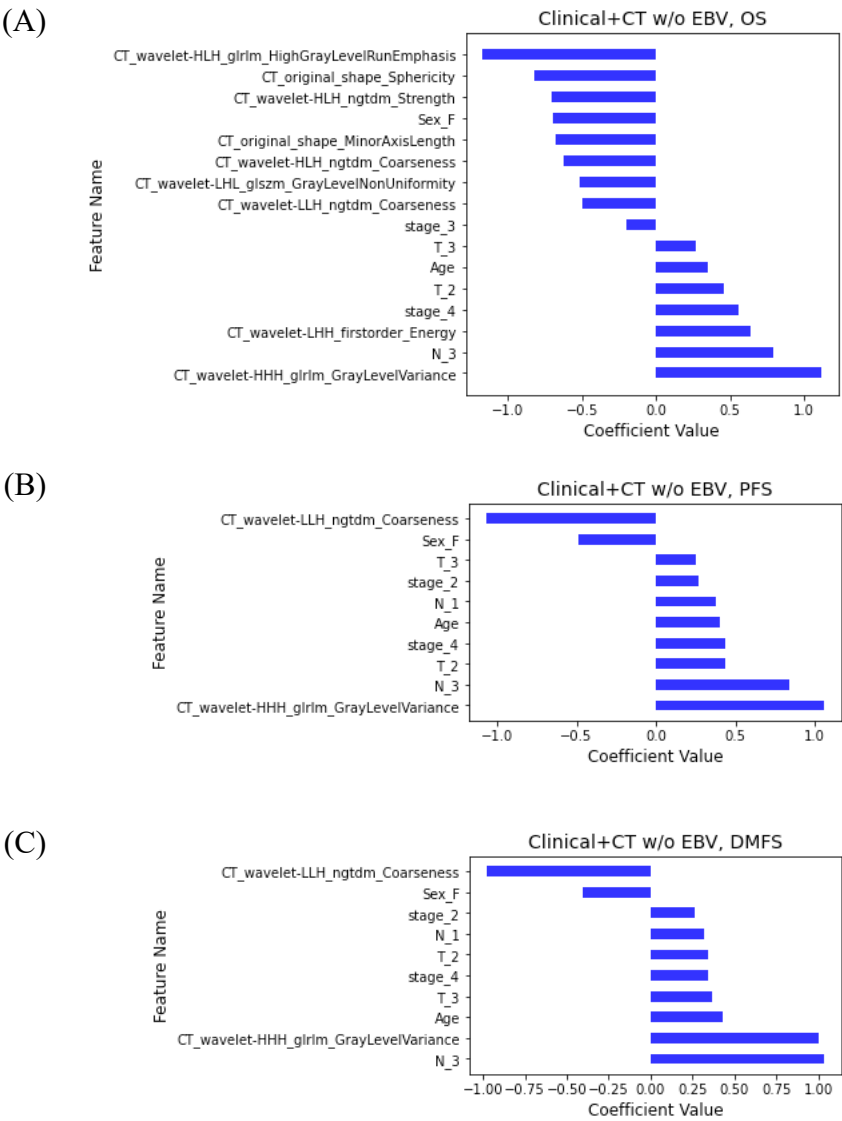

Supplement: S5 Fig — (A) overall survival (OS), (B) progression-free survival (PFS), and (C) distant metastasis-free survival (DMFS) in patients with nasopharyngeal carcinoma. (PDF) [file pone.0298111.s005.pdf]

S6 Fig

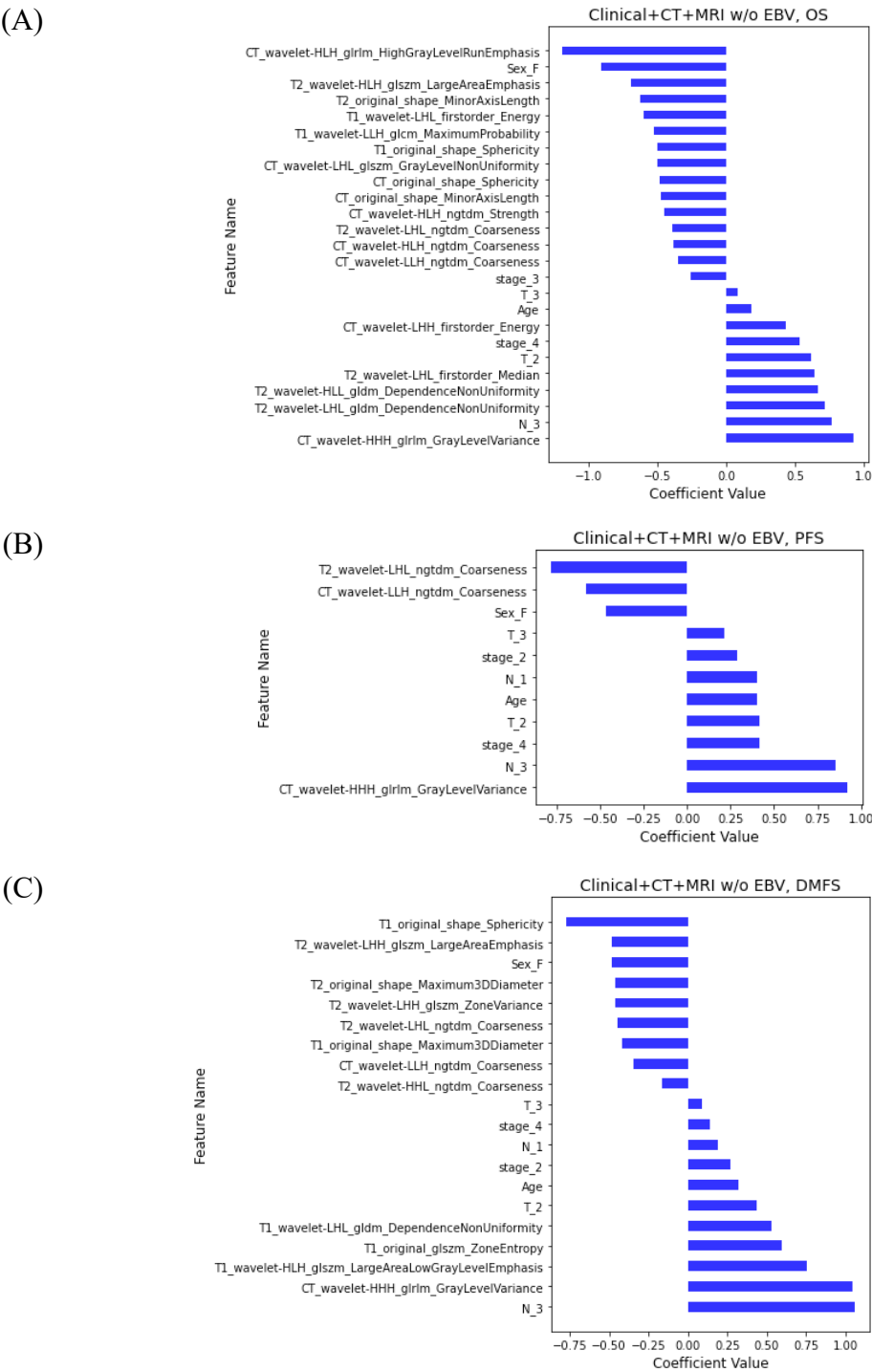

Supplement: S6 Fig — (A) overall survival (OS), (B) progression-free survival (PFS), and (C) distant metastasis-free survival (DMFS) in patients with nasopharyngeal carcinoma. (PDF) [file pone.0298111.s006.pdf]
